# A *Wisp3* Cre-knockin Allele Produces Efficient Recombination in Spermatocytes during Early Prophase of Meiosis I

**DOI:** 10.1371/journal.pone.0075116

**Published:** 2013-09-10

**Authors:** Steven Hann, Laura Kvenvold, Brittney N. Newby, Minh Hong, Matthew L. Warman

**Affiliations:** 1 Orthopaedic Research Laboratories, Department of Orthopaedic Surgery, Boston Children’s Hospital, Boston, Massachusetts, United States of America; 2 Howard Hughes Medical Institute, Boston Children’s Hospital, Boston, Massachusetts, United States of America; 3 Department of Genetics, Harvard Medical School, Boston, Massachusetts, United States of America; Montana State University, United States of America

## Abstract

Individuals with the autosomal recessive skeletal disorder Progressive Pseudorheumatoid Dysplasia have loss-of-function mutations in *WISP3*, and aberrant WISP3 expression has been detected in tumors from patients with colon and breast cancer. In mice however, neither absence nor over-expression of WISP3 was found to cause a phenotype, and endogenous *Wisp3* expression has been difficult to detect. To confirm that *Wisp3* knockout mice have no phenotype and to identify potential sites of endogenous *Wisp3* expression, we generated mice with a knockin allele (*Wisp3* ^GFP-Cre^) designed to express Green Fluorescent Protein (GFP) and Cre-recombinase instead of WISP3. Heterozygous and homozygous knockin mice were fertile and indistinguishable from their wild-type littermates, confirming that mice lacking *Wisp3* have no phenotype. We could not detect GFP-expression from the knockin allele, but we could detect Cre-expression after crossing mice with the knockin allele to Cre-reporter mice; the double heterozygous offspring had evidence of Cre-mediated recombination in several tissues. The only tissue that had high levels of Cre-mediated recombination was the testis, where recombination in spermatocytes occurred by early prophase of meiosis I. As a consequence, males that were double heterozygous for a *Wisp3* ^GFP-Cre^ and a floxed allele only contributed a recombined allele to their offspring. We detected no evidence of Cre-mediated recombination in the female ovary, although when double heterozygous females contributed the reporter allele to their offspring it had recombined ~7% of the time. *Wisp3* ^GFP-Cre^ expression therefore occurs less frequently and most likely at a later stage of oocyte development in female mice compared to male mice. We conclude that although WISP3 is dispensable in mice, male mice with a *Wisp3* ^GFP-Cre^ allele (Jackson Laboratory stock # 017685) will be useful for studying early prophase of meiosis I and for efficiently recombining floxed alleles that are passed to offspring.

## Introduction

Wnt1 inducible signaling pathway protein 3 (WISP3/CCN6) is a member of the connective tissue growth factor family (CCN) of proteins [1]. The 6 CCN family members share a common structure comprised of an N-terminus signal peptide and four domains bearing homology to insulin-like growth factor binding protein, von Willebrand factor, thrombospondin 1, and cysteine-knot containing proteins [2,3]. CCN proteins are key signaling/regulatory factors involved in a wide variety of cellular processes including, cell adhesion, extracellular matrix remodeling, skeletal development, chondrogenesis, angiogenesis, wound repair, cell proliferation and tumourigenesis (reviewed in 1,4). Such a diversity of interactions is facilitated by the modular architecture of CCN proteins, whereby the 4 domains can either act individually or in multiple combinations to confer specificity [5].

*WISP3* was first identified by DNA sequence homology and subsequently found to have altered expression levels in colon cancers [3]. Further studies showed that somatic *WISP3* frameshift mutations occur frequently in mismatch repair deficient colorectal carcinomas [6,7] and *WISP3* expression is frequently reduced or lost in inflammatory breast cancers [8,9]. Inherited loss-of-function mutations in *WISP3* cause the autosomal recessive, skeletal disorder Progressive Pseudorheumatoid Dysplasia (PPD) [10–13].

Investigations into the *in vivo* role of WISP3 in PPD and, more generally, in cartilage formation/maintenance have met with little success [14,15]. Although morpholino-mediated knockdown of *Wisp3* expression in zebrafish produces a craniofacial cartilage phenotype [16], neither over-expression [15] nor mutation of *Wisp3* [14] produced any discernable phenotype in mice. *Wisp3* and *WISP3* mRNAs were detected by RT-PCR in several mouse [14] and human [3] tissues, respectively, but not by *in situ* hybridization. Immunohistochemistry has been unable to detect endogenous WISP3 protein.

To elucidate the expression pattern of *Wisp3* in mice, we generated knockin mice in which a Green Fluorescent Protein (GFP) - internal ribosomal entry site - Cre recombinase (Cre) expression cassette was inserted in-frame into the first exon of *Wisp3*, three amino acids downstream of the endogenous *Wisp3* translation initiation codon. This knockin allele, *Wisp3* ^GFP-Cre^, was designed to express GFP and Cre in place of WISP3. Here we describe the expression pattern of the *Wisp3* ^GFP-Cre^ allele.

## Materials and Methods

All animal work was performed as approved by the Institutional Animal Care and Use Committee at Boston Children’s Hospital.

### Construction of the GFP-Cre Targeting Vector and generation of Wisp3^GFP-Cre^ knockin mice

**Figure 1:**
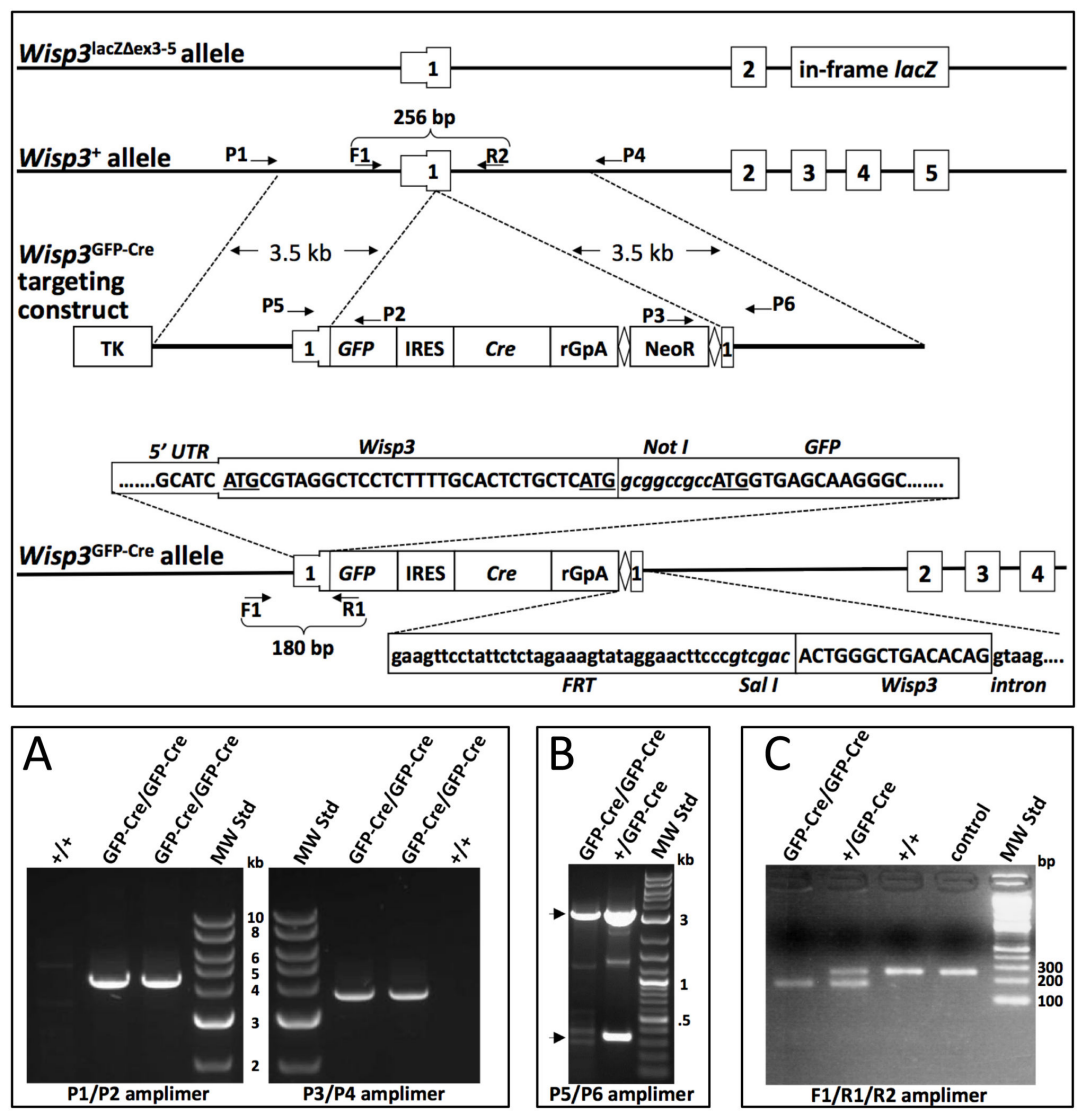
Generation of a knockin GFP-CRE allele at the *Wisp3* locus. (Upper panel) Schematic diagram of the *Wisp3* locus, the targeting vector, and the *Wisp3* ^lacZΔex3-5^ and *Wisp3* ^GFP- Cre^ alleles (not drawn to scale). Exons 1-5 are indicated as rectangles, with the 5’ untranslated region (UTR) of exon 1 a thinner rectangle. The *Wisp3* ^GFP-Cre^ targeting construct contains a thymidine kinase cassette (TK) for negative selection, an enhanced Green Fluorescent Protein (GFP) cassette, an internal ribosomal entry site (IRES), a Cre-recombinase (Cre) cassette, a polyA addition signal (rGpA), and a FRT (diamonds) flanked Neomycin resistance cassette (NeoR), all inserted into *Wisp3* exon 1. DNA sequences from the correctly targeted *Wisp3* ^GFP-Cre^ allele are shown. The two potential translation initiation codons of endogenous *Wisp3* are underlined, as is the translation initiation codon in the GFP expression cassette. NotI and SalI restriction sites are in italics. FRT sequence 3’ of the NeoR cassette, the remainder of *Wisp3* exon 1, and beginning of *Wisp3* intron 1 are indicated. Arrows indicate the locations of the PCR primers used to generate the amplimers shown in the lower panels. The P1 and P4 primers lie outside of the targeting arms (Lower panels). A) PCR amplimers demonstrating correctly targeted alleles in *Wisp3* ^GFP-Cre/GFP-Cre^ mice and their absence in wild-type mice. B) PCR amplimers demonstrating insertion of the GFP-IRES-Cre cassette into *Wisp3* exon 1 in heterozygous and homozygous knockin mice (arrows indicate the expected products from the knockin and wild-type alleles). C) PCR amplimers used to genotype wild-type, heterozygous, and homozygous knockin littermates and the *Wisp3* locus in C57BL6/J mice (control). Sizes of the molecular weight standards (MS Std) in kb or bp are indicated.

The following DNA elements were PCR amplified, subcloned, and ligated to produce a GFP-IRES-Cre-Neo targeting vector (Figure 1).

1 FRT-PGK-EM7-Neo-FRT selection cassette from pL451 [17]
2 5’ and 3’ Wisp3 targeting arms from BAC RP24_541B4 (CHORI, Oakland, CA)
3 eGFP, IRES, and Cre from pIGCN21 [18]
4 Thymidine kinase selection cassette from pL253 [17]

The full-length targeting vector was linearized and used to perform homologous recombination in V6.5 (C57BL/6 x 129S4/SvJae)F1 ES cells using positive (G418) and negative (Ganciclovir) selection. Correct targeting of ES cells was confirmed by long-range PCR (**P1** aacgccagtgctcaactccatctcac and **P2** tgctcaggtagtggttgtcg for 5’ targeting and **P3** gggaggattgggaagacaat and **P4** ccatgaaggaagccctatga for 3’ targeting), and one correctly targeted ES cell clone was introduced into C57BL6/J blastocysts to produce chimeric and then germline- transmitted *Wisp3* ^+/GFP-Cre-Neo^ mice with a mixed genetic background. The FRT-flanked Neo selection cassette was removed by crossing a *Wisp3* ^+/ GFP-Cre-Neo^ mouse with a FLP deleter mouse (B6.129S4-*Gt*(*ROSA*) *26Sor* ^*tm1(FLP1)Dym*^/RainJ; Jackson Laboratory stock # 009086). Offspring with the Neo allele excised (i.e., *Wisp3* ^+/GFP-Cre^) were used to generate the mice used in all subsequent experiments. In-frame insertion of GFP into the first exon of *Wisp3* in mice with *Wisp3* ^GFP-Cre^ alleles was confirmed by PCR (**P5** gtggagacgtggttccttgt and **P6** cacactgaagccttcttgca-3’), subcloning, and sequencing.

### Genotyping alleles at the Wisp3 and ROSA26 loci

Total DNA was extracted from mouse tail tips using the HotSHOT method [19]. Genotyping of mice with the *ROSA26* ^mTmG^ allele ((ROSA) 26Sor^tm4(ACTB-tdTomato,-EGFP)Luo^; Jackson Laboratory stock # 007576) was performed as recommended by the Jackson Laboratory for this strain. Genotyping of the *Wisp3* ^GFP-Cre^ allele was performed with a three-primer reaction (**F** cgtaggctcctcttttgcac; **R1** gaacttcagggtcagcttgc; and **R2** tgcaagaaggcttcagtgtg) designed to generate a 256 bp amplimer for the wild-type *Wisp3* allele and a 180 bp amplimer for the *Wisp3* ^GFP-Cre^ knockin allele. PCR conditions were: 94°C for 2 min followed by 30 cycles of 94°C for 30 sec, 55°C for 30 sec, and 72°C for 45 sec, followed by a 7 minute 68°C polish.

### Detecting Cre-recombination of the ROSA26^mTmG^ allele

Total DNA was extracted from individual tissues using the DNeasy blood and tissue extraction kit (Qiagen). One primer pair (**mT-F** gcaacgtgctggttattgtg and **mT-R** tgatgacctcctctcccttg) was designed to generate a 200 bp amplimer from the non-recombined *ROSA26* ^mTmG^ allele and another primer pair (**mG-F** gttcggcttctggcgtgt and **mG-R** tgctcacggatcctaccttc) was designed to generate a 376 bp amplimer from the Cre-recombined *ROSA26* ^mTmG^ allele. PCR conditions for both reactions were: 95°C for 1 min followed by 35 cycles of 94°C for 15 sec, 62°C for 30 sec, and 72°C for 1 min, followed by a 2.5 minute 72°C polish. The sensitivity of the latter primer pair for detecting Cre-recombination of the ROSA26^*mTmG*^ allele was determined by performing PCR using template genomic DNA from non- recombined mice that contained serial diluted genomic DNA from mice that had completely recombined alleles. The unmixed genomic DNAs were used as negative and positive controls for each primer pair, and the recombination assay was performed using 200 ng of template DNA.

### Tissue Collection/Preparation

Mice were euthanized immediately prior to tissue collection. For histologic analyses, dissected tissues were washed thrice in 1x phosphate buffered saline (PBS) and fixed in 4% paraformaldehyde (USB) in PBS at 4^°^C overnight. Tissues were then washed once with 1x PBS and cryoprotected in 30% sucrose in PBS at 4^°^C. Samples were embedded in OCT (Tissue-Tek) and stored at -80^°^C. Eight to 10 µm sections were cut onto glass slides, washed thrice in 1x PBS, and treated with DAPI staining solution (Molecular Probes) and Fluromount-G (Southern Biotech).

Fluorescence microscopy was performed on a Nikon Eclipse 80i fitted with Nikon Digital Sight DS-Ri1 and Photometrics Coolsnap HQ2 cameras (DAPI, FITC, Cy5 and Cy3 filters).

Images were captured using NIS-Elements AR 3.10 software, with channel and capture parameters standardized.

For DNA and RNA extraction, tissues were washed thrice in 1x PBS and placed directly in Allprotect Tissue Reagent (Qiagen) or RNAlater (Invitrogen) at 4^°^C for a minimum of 24 hrs. DNA and RNA were extracted using either DNeasy Blood & Tissue Kit (Qiagen) or Trizol (Invitrogen) following the manufacturers’ recommendations.

### RT-PCR of Wisp3

Mouse RNA’s were bought from Ambion (AM5730G), Zyagen (MR-108, MR-109) and BioChain (R4334566). Reverse transcription was performed using Ready-To-Go You-Prime First-Strand Beads (GE Healthcare) with 2 µg RNA and OligoDT(20) primers (Invitrogen).

PCR was performed using Taq-pro Complete (Denville scientific) with *Wisp3* specific, exon/intron spanning, primers (**ExF** tgtggcagttggatgtgagt and **ExR** cccggttagaaattcccatt). Non- reverse transcribed RNAs from the stocks served as negative controls for genomic DNA contamination. PCR conditions were: 94°C for 2 min followed by 35 cycles of 94°C for 30 sec, 55°C for 30 sec, and 72°C for 45 sec, followed by a 7 minute 68°C polish.

## Results and Discussion

### Creation of Wisp3^GFP-Cre^ knockin mice

Figure 1 depicts the targeting strategy used to generate the *Wisp3* ^GFP-Cre^ allele. Mouse *Wisp3* has 2 potential translation initiation codons in exon 1. We used a *NotI* restriction site to add three alanine residues between the second translation initiation codon of *Wisp3* and the translation initiation codon of GFP (Figure 1). Depending upon which of the 3 potential translation initiation codons was used by the knockin allele, the GFP protein product would contain 14, 4, or 0 additional amino acid residues at its amino terminus. We confirmed correct targeting by long-range PCR (Figure 1) and sequencing (data not shown), and excised the Neo- cassette with a Flippase expressing mouse. After confirming the Neo-cassette had been excised, we intercrossed *Wisp3* ^+/GFP-Cre^ mice and produced *Wisp3* ^+/+^, *Wisp3* ^+/GFP-Cre^, and *Wisp3* ^GFP-Cre/GFP-Cre^ offspring (Figure 1) at the expected Mendelian frequencies. *Wisp3* ^+/GFP-Cre^ and *Wisp3* ^GFP- Cre/GFP-Cre^ mice were indistinguishable from their wild-type littermates when followed to >12 months-of-age, and they were fertile. Seventeen matings between *Wisp3* ^GFP-Cre/GFP-Cre^ mice produced an average of ~9 pups/litter and a male: female pup ratio of 1.07.

We previously generated a mutant allele of *Wisp3*, *Wisp3* ^lacZΔex3-5^, by replacing exons 3 -5 with a lacZ reporter and a Neomycin selection cassette (Figure 1) [14]. This mutant allele was designed to produce a mutant allele of mouse *Wisp3* that was comparable to several homozygous deletion and nonsense *WISP3* mutations that cause Progressive Pseudorheumatoid Dysplasia in humans [10,12]. These homozygous *Wisp3*-mutant mice also appeared normal and were fertile on two different genetic backgrounds (129SvEv and C57BL/6) [14]. Thus, the inability of two different *Wisp3* loss-of-function alleles to recapitulate the phenotype of Progressive Pseudorheumatoid Dysplasia on different genetic backgrounds argues strongly against WISP3 function in the skeleton being conserved between humans and mice. We cannot preclude the possibility that another murine CCN family member is compensating for WISP3 deficiency. To date, only *Wisp1*/*Wisp3* double knockout mice have been studied; they are indistinguishable from *Wisp1* knockouts alone (personal communication from Professor Karen Lyons, UCLA, Los Angeles, CA).

### Comparing sites of endogenous Wisp3 expression to sites of GFP and Cre expression from the Wisp3^GFP-Cre^allele

**Figure 2:**
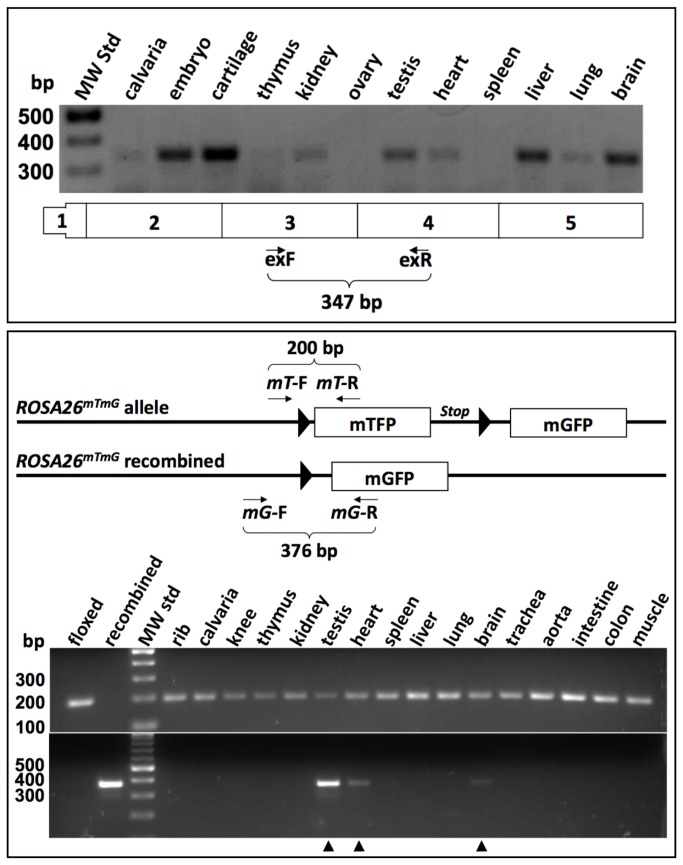
Endogenous *Wisp3* expression and *Wisp3* ^*GFP-Cre*^ expression are not identical. (Upper panel) RT-PCR amplimers indicating the presence of *Wisp3* transcript in total RNA from a several different mouse tissues. A schematic of *Wisp3* mRNA is indicated (not drawn to scale) along with the locations of the intron-spanning PCR primers and the expected amplimer size for correctly splice mRNA (Lower panel). A schematic of the *ROSA26* ^mTmG^ allele before and after Cre-recombination (not drawn to scale) along with the locations of the PCR primers and the expected amplimer sizes for the non-recombined and recombined alleles. PCR amplimers indicating non-recombined *ROSA26* ^mTmG^ DNA (upper gel) in all tissues and Cre-mediated recombination (lower gel arrowheads) in testis, heart, and brain recovered from *Wisp3* ^+/GFP- Cre^;*ROSA26* ^+/mTmG^ mice (floxed and recombined template DNA serves as controls for the two primer pairs). Note that there is poor correlation between endogenous *Wisp3* expression (upper panel) and *Wisp3* ^GFP-Cre^ activity (lower panel).

**Figure 3:**
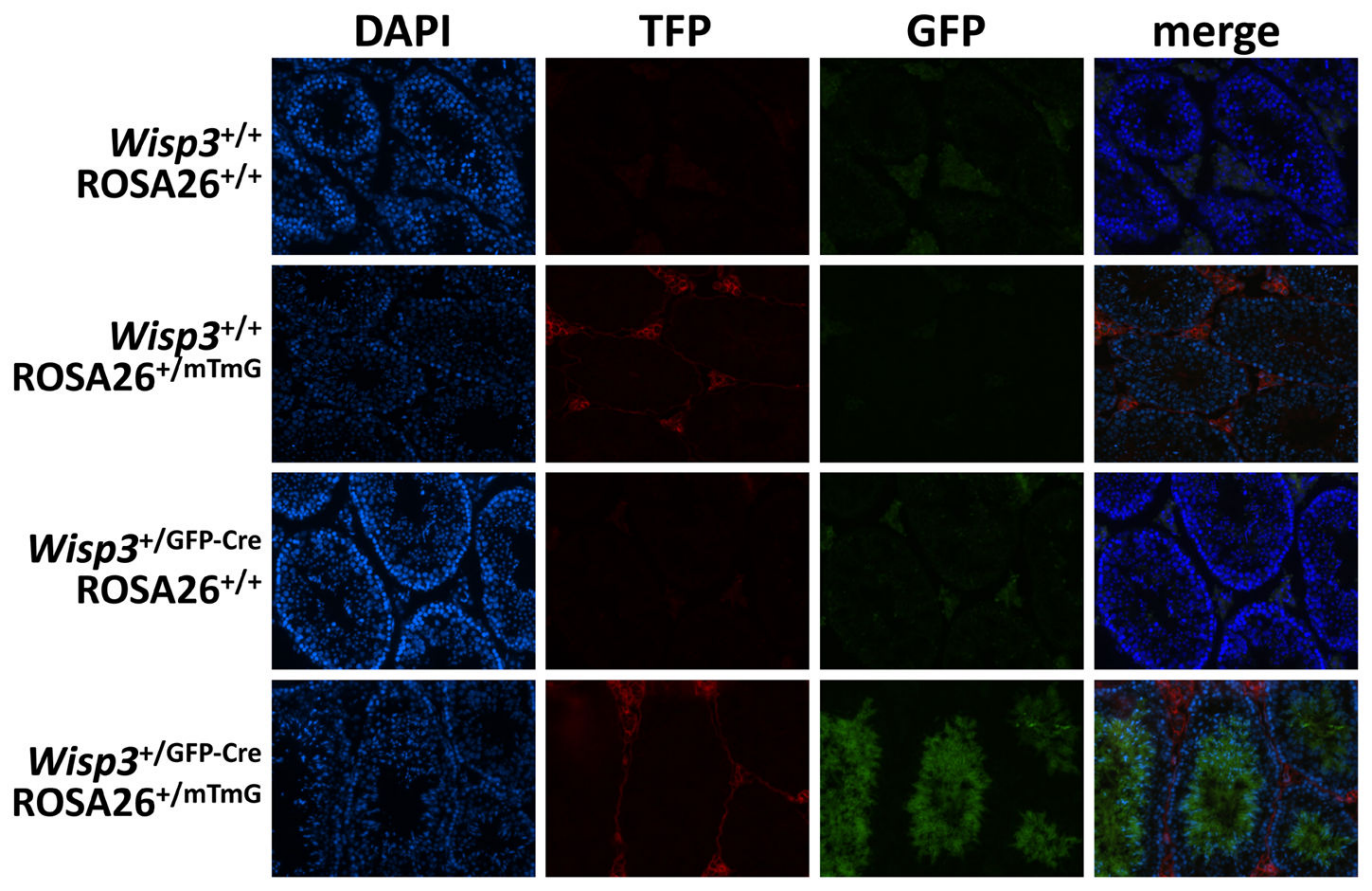
The *Wisp3* ^GFP-Cre^ allele is expressed during spermatogenesis. Fluorescence microscope images of seminiferous tubules from adult male mice that are wild-type (Wisp3^+/+^) or heterozygous knockin (*Wisp3* ^+/GFP-Cre^) at *Wisp3*, and wild-type (ROSA26^+/+^) or heterozygous knockin (*ROSA26* ^+/mTmG^) at *ROSA26*. Cell nuclei are imaged with DAPI dye. Autofluorescence in the GFP channel is observed in *Wisp3* ^+/+^;*ROSA26* ^+/+^ and *Wisp3* ^+/GFP-Cre^;ROSA26^+/+^ mice. Tomato Fluorescent Protein (TFP) is observed in *Wisp3* ^+/+^;*ROSA26* ^+/mTmG^ mice and in the non- spermatocyte/spermatid cells of *Wisp3* ^+/GFP-Cre^;*ROSA26* ^+/mTmG^ mice. Green Fluorescent Protein (GFP) is observed in spermatocytes and spermatids, but not in spermatogonia, of *Wisp3* ^+/GFP- Cre^;*ROSA26* ^+/mTmG^ mice.

*Wisp3* expression is poorly represented in expressed sequence tags (EST) and RNA sequencing (RNA-seq) data sets, and has not been reliably detected by *in situ* hybridization [14]. Therefore, to determine which mouse tissues express *Wisp3* mRNA, we performed RT-PCR using commercially available total RNA from multiple mouse tissues. We were able to amplify spliced *Wisp3* RNA from several tissues, including cartilage, kidney, testis, heart, liver, and brain (Figure 2). To determine whether the *Wisp3* ^GFP-Cre^ allele expressed GFP in these tissues, we examined frozen sections from *Wisp3* ^GFP-Cre/GFP-Cre^ mice by fluorescence microscopy. We were unable to detect GFP expression from the *Wisp3* ^GFP-Cre^ allele in any tissue (Figure 3 and data not shown). We next determined whether Cre was expressed from the *Wisp3* ^GFP-Cre^ allele by crossing *Wisp3* ^+/GFP-Cre^ mice with *ROSA26* ^mTmG/mTmG^ mice and looking for evidence of Cre-mediated recombination at the *ROSA26* locus in multiple tissues by PCR and by fluorescence microscopy. We first determined that our PCR protocol could detect ~ 0.1% recombination of the *ROSA26* ^mTmG^ allele by mixing genomic DNA from mice in which the *ROSA26* ^mTmG^ allele was intact or fully recombined (data not shown). We then used this assay to identify which tissues in *Wisp3* ^+/GFP-Cre^; *ROSA26* ^+/mTmG^ double heterozygous mice had evidence of Cre-mediated recombination, and found evidence of recombination in testis, heart, and brain. Because the PCR assay was not quantitative, we also examined these same tissues for recombination using fluorescence microscopy; cells with a *ROSA26* ^mTmG^ allele that had not recombined have membranes that fluoresce red, while cells with a Cre-recombined *ROSA26* ^mTmG^ allele have membranes that fluoresce green. Testis was the only tissue in which fluorescence microscopy revealed Cre-mediated recombination of the *ROSA26* ^mTmG^ allele (Figure 3 and data not shown).

Although the Cre activity we observed for the *Wisp3* ^GFP-Cre^ allele in testis was consistent with the endogenous *Wisp3* expression we observed in this tissue by RT-PCR, and other investigators observed in human testis [3], there was no correlation between Cre activity and *Wisp3* expression in other tissues. For example, cartilage, liver, and kidney had detectable *Wisp3* expression by RT-PCR but no evidence of Cre activity in the reporter mice. One explanation for why *Wisp3* expression may have been detected by RT-PCR, while Cre-mediated recombination was not, is that only a tiny fraction (< 0.1%) of cells in these tissues express *Wisp3* but at very high levels. These few high-expressing cells could make transcript detectable by RT-PCR in a total tissue RNA pool, but since only a very small fraction of cells actually express Cre from the *Wisp3* ^GFP-Cre^ allele, Cre-mediated recombination occurred too infrequently to be detected with our recombination assay. The alternative and more likely explanation is that Cre-expression from the *Wisp3* ^GFP-Cre^ allele does not truly mirror endogenous *Wisp3* expression. That we could not find cells, other than spermatocytes and their descendents in the testis, expressing membrane bound GFP in the *Wisp3* ^+/GFP-Cre^; *ROSA26* ^+/mTmG^ double heterozygous mice supports this latter explanation.

### The Wisp3^GFP-Cre^ allele is highly efficient at producing Cre-mediated recombination during early prophase of male meiosis I

**Figure 4:**
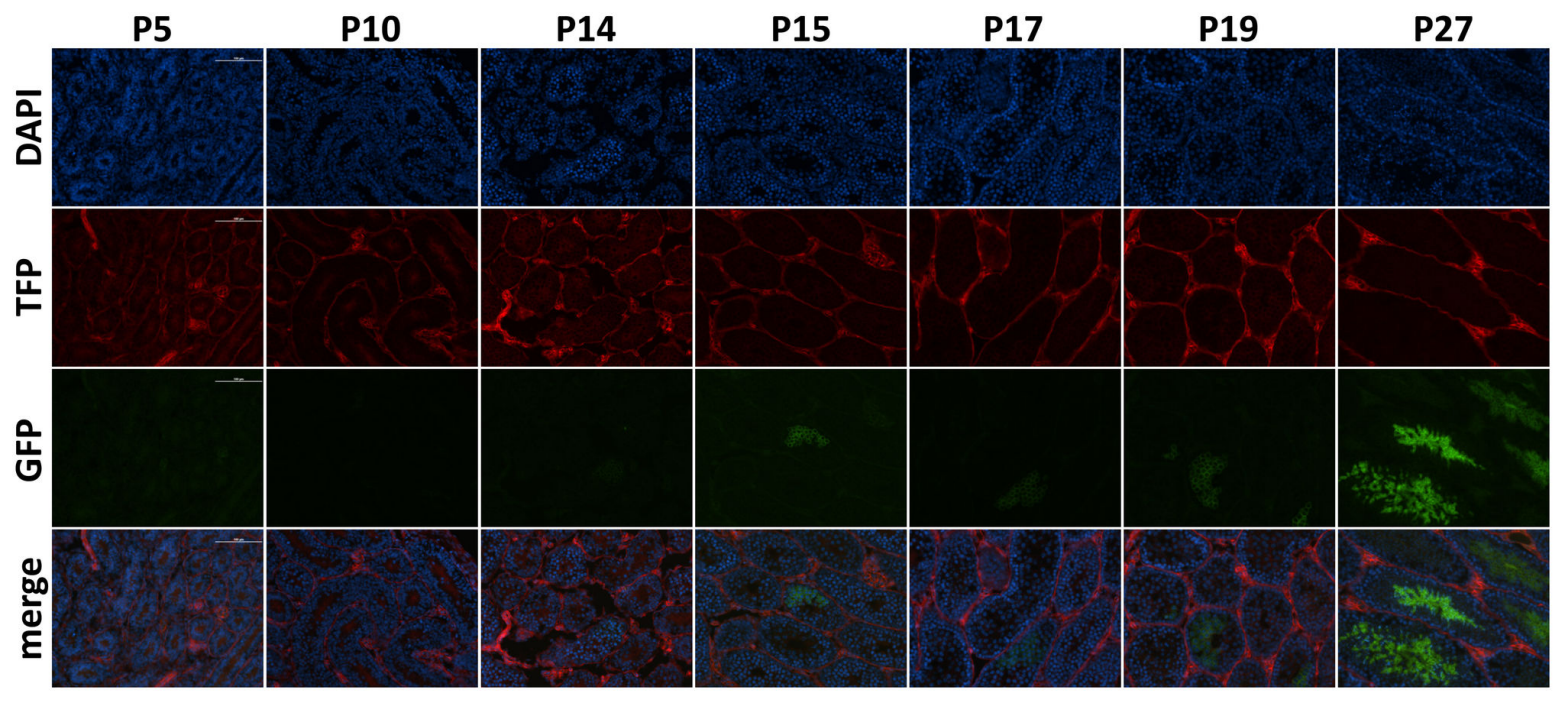
*Wisp3* ^GFP-Cre^ activity occurs in spermatocytes early during meiosis I. Fluorescence microscope images of seminiferous tubules from *Wisp3* ^+/GFP-Cre^;*ROSA26* ^+/mTmG^ male mice at 5, 10, 14, 15, 17, 19 and 27 days-of-age (P5 – P27). Cell nuclei are imaged with DAPI dye. Spermatocytes expressing membrane bound Green Fluorescent Protein (GFP) instead of Tomato Fluorescent Protein (TFP), which indicates that Cre-mediated recombination has occurred, become visible by P15 and increase in abundance with age. Germ cells and spermatogonial cells, which reside near the periphery of seminiferous tubules, do not express GFP. The location of the GFP expressing cells in the seminiferous tubules, coupled with PCR evidence of recombination by P10 (data not shown), suggests that the *Wisp3* ^GFP-Cre^ allele is expressed in spermatocytes between the leptotene and pachytene stages of male meiosis I.

The strong Cre-activity we observed in the testis of *Wisp3* ^+/GFP-Cre^ mice led us to determine the efficiency of this allele in producing germline recombination. We bred two *Wisp3* ^+/GFP-Cre^; *ROSA26* ^+/mTmG^ males to wild-type females. Of 186 offspring generated from these crosses, 85 mice inherited the *ROSA26* ^mTmG^ allele and this allele had recombined in all (100%). To identify the earliest stage during spermatogenesis at which Cre activity became detectable, we collected testes from male *Wisp3* ^+/GFP-Cre^; *ROSA26* ^+/mTmG^ mice at different postnatal ages and examined them for evidence of Cre-activity by PCR and fluorescence microscopy (Figure 4). By fluorescence microscopy, Cre-recombination of the *ROSA26* ^mTmG^ allele was apparent in the seminiferous tubules of 15-day-old (P15) mice. Our PCR-based assay was able to detect evidence of Cre-mediated recombination in testis as early as P10 (data not shown). This timeframe coincides with the onset of early prophase of meiosis I during mouse spermatogenesis [20].

We performed the converse experiment by breeding wild-type males and three *Wisp3* ^+/GFP-Cre^; *ROSA26* ^+/mTmG^ females. Of 157 offspring, 72 inherited the ROSA26^mTmG^ allele and this allele had recombined in 5 of the 72 mice (~7%). All three females had pups with a recombined allele. Recombination appears to have occurred during female gametogenesis, since none of the 5 pups had evidence of somatic mosaicism for the non-recombined and recombined alleles by PCR (data not shown). We examined the ovaries of the *Wisp3* ^+/GFP-Cre^; *ROSA26* ^+/mTmG^ dams by fluorescence microscopy to look for evidence of Cre-mediated recombination in oocytes but found none (data not shown). Most oocytes in fertile female mice are arrested at the diplotene stage of prophase I [21,22]. Since that stage of oocyte arrest is later than the stage in meiosis in which *Wisp3* ^+/GFP-Cre^-mediated recombination had already occurred in males, it appears as though *Wisp3* ^+/GFP-Cre^ expression commences later and less often during female meiosis compared to male meiosis.

Several transgenic and knockin mice express Cre-recombinase in male spermatogonia, spermatocytes, and/or spermatids (reviewed in 23,24). Mice that express Cre-recombinase during spermatogenesis or oogenesis, and that are heterozygous for a floxed allele, have great utility in generating recombined alleles in their progeny. Male *Wisp3* ^GFP-Cre^ mice, which we have donated to The Jackson Laboratory (stock # 017685), should be particularly useful for this purpose since they are healthy, fertile, and highly efficient Cre-deleters in early spermatocytes. Also, since the *Wisp3* ^GFP-Cre^ allele is a knockin, rather than a transgene, its chromosomal location is known (Chr10:39150971-39163794; in the GRCm38/mm10 genome assembly) and it is unlikely to be subject to epigenetic silencing. In addition to transmitting recombined alleles to progeny, *Wisp3* ^GFP-Cre^ mice can be used to specifically determine the consequence of altering gene expression in spermatocytes during meiosis I. The *Wisp3* ^GFP-Cre^ allele produces minimal Cre-recombinase activity in cells/tissues other than spermatocytes, and thus *Wisp3* ^GFP-Cre^ mice are unlikely to have significant off-target effects. This contrasts with transgenic strains containing Cre-drivers that are active during male meiosis I, such as Pgk2-Cre [25,26], Hspa2- Cre [27,28], and Synapsin-Cre [29]. These strains are efficient Cre-deleters during paternal transmission but they exhibit significant Cre-activity in multiple tissues during embryogenesis and post-natally, which can limit their usefulness in studying male spermatogenesis.

## Conclusion

The *Wisp3* ^GFP-Cre^ allele produces extremely efficient Cre-recombinase activity in male spermatocytes by early prophase of meiosis I, and minimal recombination in other tissues. Thus, males with a *Wisp3* ^GFP-Cre^ allele and floxed alleles at other loci can be used to study the roles of these other loci during male meiosis I, without causing significant off-target effects. Importantly, by driving complete recombination in male gametes, the Wisp3^*GFP-Cre*^ allele should be very useful for converting a floxed allele to a recombined allele in offspring. *Wisp3* ^GFP-Cre^ mice have been donated to the Jackson Laboratory (stock # 017685). 

Three reporters (LacZ, GFP, and CRE) have been knocked into the *Wisp3* locus, and none have mirrored the *Wisp3* expression pattern suggested by RT-PCR. Consequently, the *Wisp3* ^GFP-Cre^ allele may not inform us regarding the endogenous expression pattern of *Wisp3*. Furthermore, the lack of a phenotype in *Wisp3* ^GFP-Cre/GFP-Cre^ mice indicates that the mice do not recapitulate the phenotype of WISP3 deficiency in humans and are unlikely to inform us regarding the pathogenesis of human Progressive Pseudorheumatoid Dyplasia. Despite their lack of utility in teaching us about PPD, male mice with *Wisp3* ^GFP-Cre^ alleles are excellent “Cre- deleters” and male and female mice offer a new means for dissecting spermatogenesis and oogenesis. As with any genetically modified allele, it is possible that the Wisp3^GFP-Cre^ allele, alone, could produce a phenotype depending on the mouse’s genetic background. Investigators should consider this possibility when using the Wisp3^GFP-Cre^ allele in their experiments.
